# Creation and evaluation of a participatory child abuse and neglect workshop for medical students

**DOI:** 10.1186/s12909-022-03837-2

**Published:** 2022-11-16

**Authors:** Christos Giannakas, Aspasia Manta, Maria Effrosyni Livanou, Vasiliki Daniil, Angeliki Paraskeva, Maria-Konstantina Georgiadou, Nefeli Griva, Vassiliki Papaevangelou, Maria Tsolia, John M. Leventhal, Alexandra Soldatou

**Affiliations:** 1 Medical School, National and Kapodistrian University of Athens, Athens, Greece; 2 3rd Department of Pediatrics, National and Kapodistrian University of Athens, Rimini 1, 124 62 Chaidari, Greece; 3 2nd Department of Pediatrics, National and Kapodistrian University of Athens, Thivon and Levadeias, Goudi 11527, Athens, Greece; 4 Department of Pediatrics, Yale School of Medicine, 333 Cedar St, CT 06510 New Haven, USA

**Keywords:** Child maltreatment, Medical curriculum, Active learning, Medical education, Peer-to-peer teaching, Role-playing

## Abstract

**Background:**

Since child abuse and neglect (CAN) is prevalent worldwide, medical students should acquire basic knowledge, skills, and confidence in identifying and addressing CAN. Although significant educational efforts have been previously described, none has focused on using participatory methods to teach medical students CAN.

**Purpose:**

To: 1) develop a participatory educational workshop in CAN for medical students, 2) gather, train, and establish a peer-to-peer teaching group, and 3) assess the effectiveness of the workshop in gain of knowledge and improvement of self-confidence for participants.

**Methods:**

A two-hour workshop was created with role-playing, the use of mannikins and peer-to-peer teaching. A 15-item knowledge and a 9-item self-confidence questionnaire were used before, right after, and six months after each workshop.

**Results:**

Nine workshops in two academic pediatric departments with a total attendance of 300 6th year medical students were conducted. For the 69 students who completed the questionnaires at all three times, there were statistically significant gains in knowledge right after (*p* < .001) and six months after (*p* < .0001) the workshops. Similarly, self-confidence increased right after (*p* < .0001) and six months after (*p* < .001) the workshops. Self-selection bias testing indicated that these 69 students who completed all three questionnaires were representative of those who completed the pre-testing and the testing right after.

**Conclusions:**

We successfully established a peer-to-peer teaching group to conduct nine participatory workshops that improved the participants’ knowledge and self-confidence in CAN. This feasible and novel active learning approach may help address inadequacies in medical curricula.

**Supplementary Information:**

The online version contains supplementary material available at 10.1186/s12909-022-03837-2.

## Introduction

Early detection and evidence-based management of child abuse and neglect (CAN) by health care professionals can be crucial for the child’s long-term health, physical integrity or even life [1]. Despite improvements in child protection in many countries, most victims of CAN remain without diagnosis, protection and treatment, confirming the iceberg phenomenon [2]. The decline in child death rates is still very low, with infants at the highest risk of CAN-related fatalities [3]. To stress the need for immediate action, the WHO has declared the issue a Public Health Priority [3]. Prevention of fatal CAN could contribute to the attainment of the Millennium Development Goal 4 of reducing child mortality in children under five years of age [4].

To address the lack of knowledge, skills, and confidence in identifying and addressing CAN reported by medical students and healthcare professionals worldwide [5–14], significant efforts have been made in many countries including the United States, the United Kingdom, Greece and Taiwan, regarding CAN education, with encouraging results [15–21]. Teaching methods and educational interventions described in the literature mostly involve conventional didactic lectures [17–21], handing out protocols [15, 18], decisional flow charts and self-instructional kits with self-evaluation activities [20], case presentations and discussions with experts [15, 18, 21], direct participation in patient evaluation [21], videos [15, 21] and audiotapes [19], e-learning modules [17, 19, 22], cards-illustrations and written scenarios [20] or virtual patients [19]. There is only one study describing a simulated encounter of nursing students with a person acting as the mother of an injured infant [23].

Interactive case-based workshops on CAN recognition and reporting for healthcare professionals have been previously shown to be effective and have stronger and longer lasting effects compared to didactic lectures [15–17, 20, 24]. Data suggest that active participation during educational sessions enhances the participants’ ability to learn [25] as adult learners learn best in an interactive training setting [26]. In addition, the effectiveness of peer teaching and its multiple potential benefits for learners and teachers have also been previously shown [27].

Despite the need to adopt novel and effective educational approaches, to the best of our knowledge, there are no articles on the development and evaluation of simulation-based training methods for medical students with role-playing, the use of mannikins, and peer-to-peer teaching in CAN. Therefore, to bridge this gap, the aims of this study were to 1) develop a participatory educational workshop in CAN for medical students, 2) gather, train and establish a peer-to-peer teaching group, and 3) assess the effectiveness of the workshop in gain of knowledge and improvement of self-confidence for participants.

## Methods

### Structure of workshops

The study was conceived and designed by two medical students in collaboration with a pediatric faculty member at NKUA, who is also a CAN expert in Greece. A two-hour workshop was created consisting of two parts. The first part was introductory and acted as a “presimulation briefing”, as described by Rudolph et al. [28]. It included a 15-min presentation of the most crucial characteristics of CAN, as well as the following five tools that can be used for the diagnosis: 1) Leventhal’s triangle, a graphic representation of the three diagnostic possibilities in every pediatric injury (i.e. accident/medical problem—abuse—neglect) [29], 2) a template of a medical history timeline depicting the most important events to focus on, 3) red flags in the medical history and physical examination suggestive of CAN, including a picture of the TEN-4 bruising rule [30], 4) an outline of the child abuse workup, including the skeletal survey [31], and 5) a universal evidence-based hospital procedures protocol for the identification and management of suspected CAN, created by CAN experts.

The second part of the workshop was participatory according to the basic principles of “simulation-based medical education (SBME)” [32]. Volunteer medical students were recruited and trained to act as the parent of a pediatric patient in the form of simulation mannikins. Five mannikins were created using plain dolls sold commercially. Real pictures of physical findings were printed and glued onto the dolls. Participants were divided into five groups (5 – 8 students), each one of which approached a different scenario. Five clinical scenarios were used (Table 1) that were based on real cases and adapted to exclude any potential identifying features in observation of General Data Protection Regulation (GDPR). Although it may be challenging to avoid the interference of any personal stereotypes and opinions, the scenarios were structured in a way that facilitated objective data gathering and a universal diagnostic process.

**Table 1:** Brief descriptions of the workshop scenarios

Scenario	Summary	Leventhal’s Triangle
#1	4-month-old male with a transverse fracture of left femur. Ethnic minority mother claimed she was holding the infant around her waist, when she fell from a damaged chair onto the floor onto the infant’s leg, while visiting a friend in a slum area. She sought medical help immediately. There were no other findings.	
#2	3-year-old female presented to the ER with fever. An iron burn on the frontal aspect of the right thigh and a superficial non-patterned burn on the right shin were noted. Single unemployed mother claimed the toddler got dangling cord of hot iron caught around her leg and subsequently landed on her thigh several days prior. Two previous hospitalizations due to ingestions. Τwo younger siblings.	
#3	3-month-old male with a 15-day history of multiple episodes of loss of consciousness. On exam bruising and abrasions noted on his head. Caregivers claimed he was struck with a shoe by his 17-month-old sibling. Mother was a nurse on parental leave, father worked at a local municipality. Head MRI revealed subacute and chronic subdural hematomas.	
#4	11-month-old female with multiple bruises of different color on the torso. Parents claimed they noticed the bruises a few hours prior to presentation. No history of trauma. Both parents worked in the private sector. Infant attended private daycare in the mornings. Bleeding diathesis workup negative.	
#5	2.5-year-old female with perianal and genital warts. Born by C-section. Father and 11-year-old brother recently treated for genital warts. Mother never had genital warts and had recent negative genital HPV testing. Toddler exclusively cared for by mother. There was a previous community report of abuse. Local social services reported the toddler’s house was clean and tidy.	

Participants were required to address the parents, take the medical history, examine the “patient” and request laboratory tests and imaging studies. Based on the above, each group decided on the most probable diagnosis, depicted as a point on Leventhal’s Triangle along the continuum between accident/medical condition, abuse, and neglect. The group also determined the need to report if the point along one side of the Triangle fell below the horizontal line that suggested maltreatment. The faculty member with two volunteer medical students oversaw all groups and provided the results of laboratory tests and imaging upon request.

In the end, a representative from each group presented the case, the diagnosis reached and the decision to report or not. All participants were asked to challenge and comment on each group’s diagnostic processes. This “postsimulation debriefing” [28] was useful for participants to make the most of the intervention and consider how to apply the skills learned in future clinical practice. Legal mandates and tips on communication with parents were also discussed.

Throughout the workshop, participants were encouraged to express their opinions and ask questions, while the teaching group aimed to generate discussions and provide tools, instead of giving “correct” answers.

### Recruitment and training of actors

In the beginning of the study, five actors and two coordinators were involved. The actors were medical students from NKUA. The coordinators were a medical graduate and the Pediatric Faculty. An active campaign among junior medical students was initiated to recruit volunteers talented in acting, who could replace senior students once they entered their clerkships. Fifteen new actors were recruited. All volunteer actors were trained on the scenarios initially by the pediatric faculty, and by their peers thereafter. They were also encouraged to observe one workshop prior to acting. Newly recruited actors were always overseen by more experienced actors, who moved into a coordinator role. A minimum number of volunteers was defined to ensure small student groups and to cover for absences.

### Pilot workshop

A pilot workshop was conducted. Revisions were based on the volunteer actors’ and participants’ feedback.

### Setting of workshops

The workshops were incorporated in the pediatric teaching curriculum during two academic years, from September 2018 to June 2020. Participants were recruited from two of three Pediatric Departments of the NKUA. Specifically, 6^th^ year medical students attended the workshops during their mandatory clinical rotation in Pediatrics in these two departments. Students who gave consent to participate in the evaluation of the workshops provided an email address.

### Evaluation questionnaires

First, participants were asked to indicate prior participation in any educational session regarding CAN at medical school. A questionnaire consisting of 15 knowledge and nine self-assessment questions was created. Multiple-choice knowledge questions were adapted from a questionnaire by Johnson (Additional file 1) [18]. Self-assessment questions were adapted from the CAN reporting self-efficacy (CANRSE) instrument (Additional file 2) [33]. Each correct answer in the knowledge questionnaire was given one point, while self-assessment questions were self-scored in a scale from 0 to 10, 0 being “not at all confident” and 10 being “extremely confident”. The participants were asked to complete the same questionnaires before, right after, and six months after the workshop. All questionnaires were completed anonymously on Google forms via email. Completion of all three questionnaires six months after the workshop secured a certificate of participation. An optional open-ended question to add comments was placed at the end of the questionnaire to collect qualitative data.

The study protocol was approved by the Committee of Bioethics and Deontology of the NKUA Medical School (Date 19.11.2018/No 13). All participating medical students were given a code that ensured their anonymity. Participation and questionnaire completion did not affect their grade or clerkship evaluation.

### Statistical analysis

Statistical analysis was conducted using the GNU PSPP statistical package, while boxplots were created using Microsoft Office Excel. Paired sample t-tests were conducted when criteria of normality of distribution were met. Wilcoxon test for paired samples was conducted when normality criteria were not met. The possibility of self-selection bias was examined with the use of non-paired t-test to compare participants who completed all three questionnaires (before, right after, and six months after the workshop) with participants who dropped out of the research right after or six months after the workshop to establish if they were similar at baseline. We evaluated if there were differences between the participants who completed all three questionnaires and those who completed only two (before and right after) regarding their scores on the questionnaires completed after the workshop. The significance of the results was assessed using the 95% confidence interval. A two-sided probability value of *p* < 0.05 was considered statistically significant. Cohen's coefficient was used to assess the magnitude of the difference between groups.

### Qualitative data analysis

All comments provided by participants as a response to the open-ended question were reviewed by two researchers independently. Comments were grouped according to their nature (positive or negative) and the theme of the suggestions.

## Results

### Sample description

**Fig. 1:**
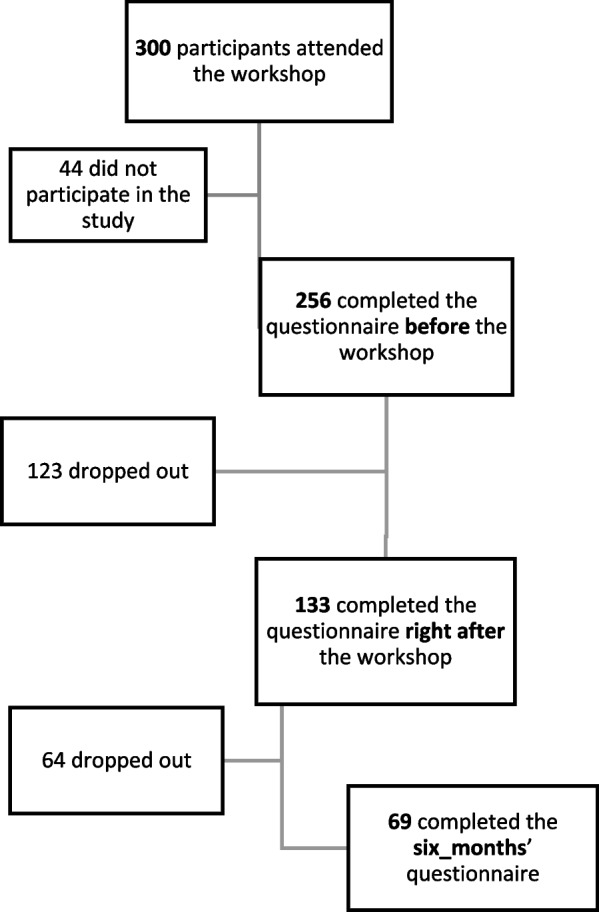
Flow-chart depicting the rate of participation and drop-out at each stage of the study

Nine workshops were conducted during the study period with a total of 300 attendees. Altogether, 256 fully completed questionnaires were collected before all nine workshops. At the end of the research, 133 paired before-right after questionnaires and 69 matched triplets of before, right after and six months after questionnaires were gathered (Fig. 1). Based on self-report, 45 of the 256 participants (17.5%) had received some form of education on CAN prior to their participation in the workshop.

### Paired-samples analysis

#### Assessment of knowledge

**Fig. 2:**
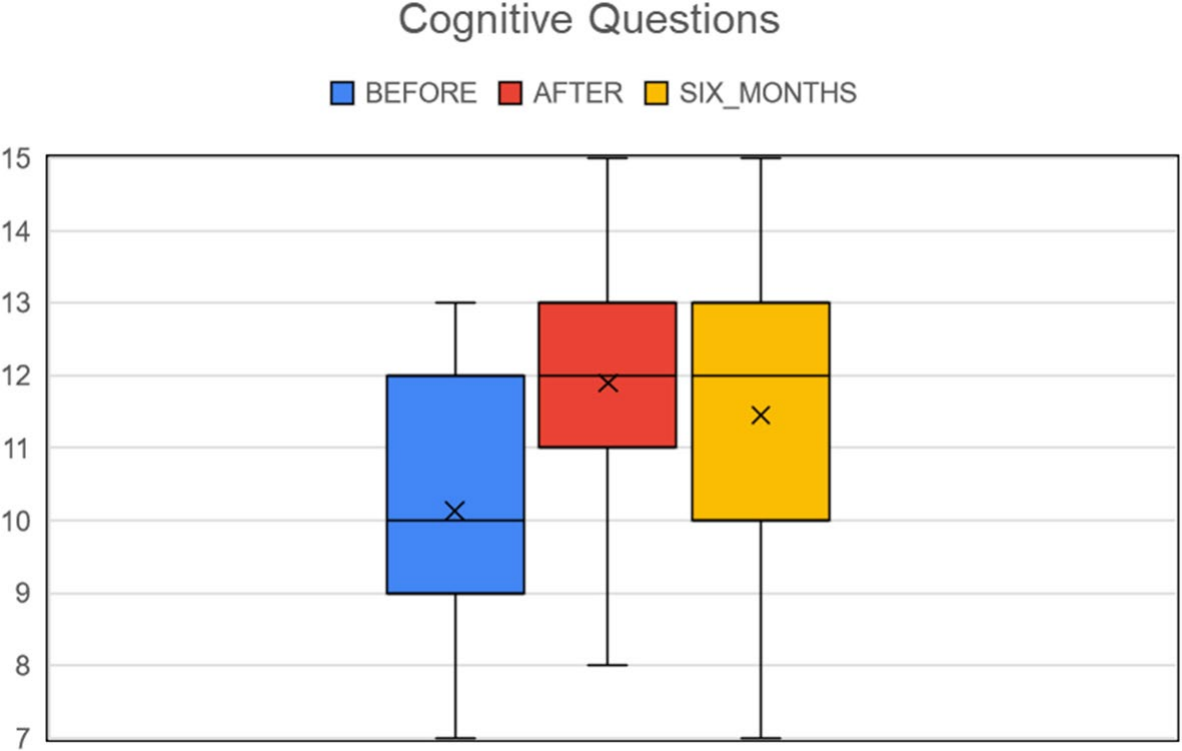
Number of correct answers to knowledge questions before, right after and six months after the workshop. Mean values are represented by x and median values by the horizontal line

In this section we present the results of the analysis of the 69 matched triplets (before, right after and six months after the workshop). The mean score on the knowledge questionnaire before, right after and six months after the workshops was 10.21 (SD ± 1.78), 11.96 (SD ± 1.53) and 11.48 (SD ± 1.81) respectively. Paired t-tests revealed a significant gain of knowledge right after the workshop compared to before (*p* < 0.001, Cohen’s d = 1.05), that was maintained six-months after the workshop (*p* < 0.0001, Cohen’s d = 0.71) (Fig. 2).

#### Assessment of self-confidence

**Fig. 3:**
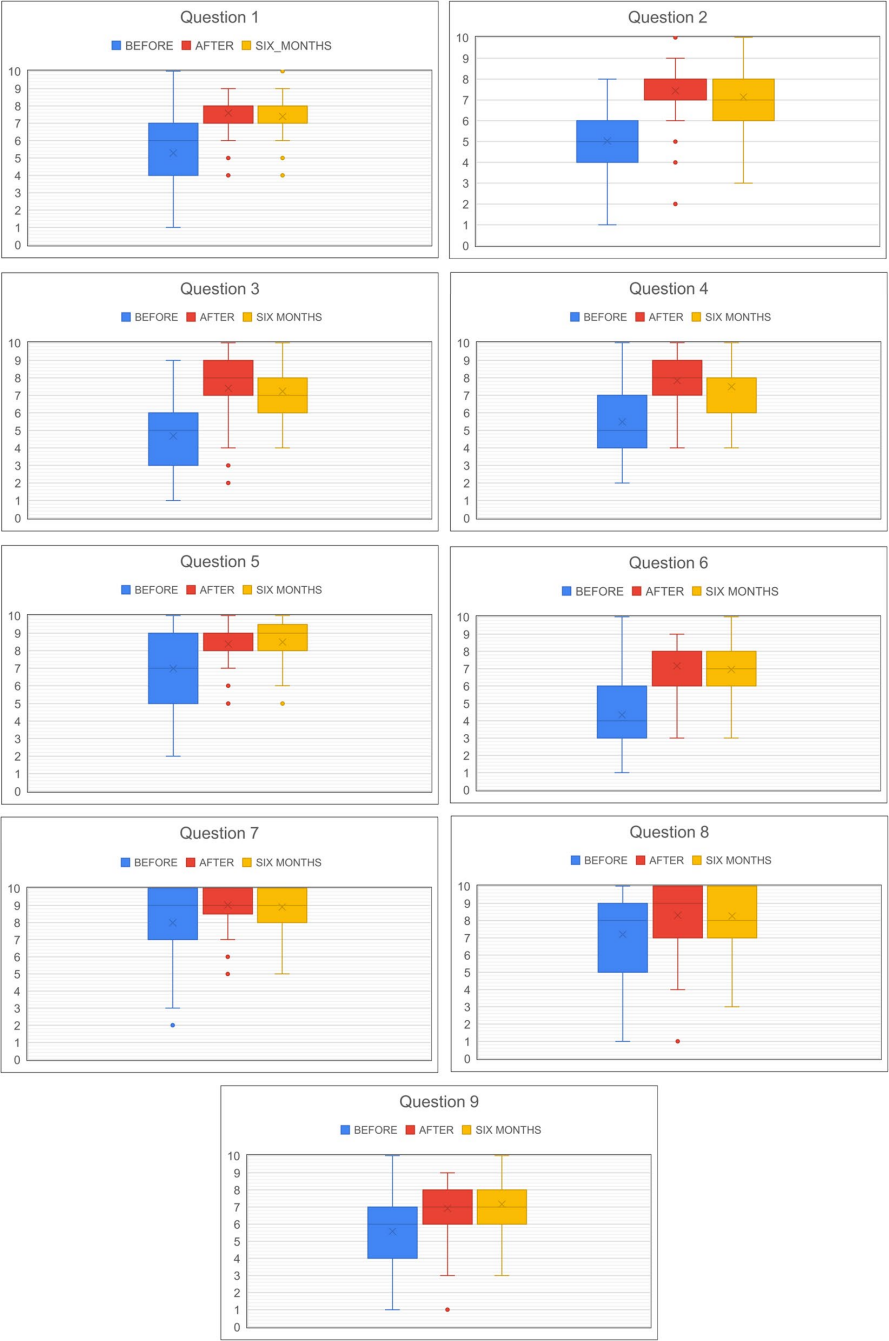
Self-confidence question scores, Questions 1–9. Mean values are represented by x and median values by the horizontal lines

For each of the nine self-assessment questions, we checked for differences in the pre-evaluation and post-evaluation scores. Wilcoxon signed-rank test for paired samples revealed a statistically significant increase in self-confidence in all nine questions (Questions 1–6, 8 and 9: *p* < 0.0001, Question 7: *p* < 0.001) (Fig. 3).

#### Testing for self-selection bias

**Fig. 4:**
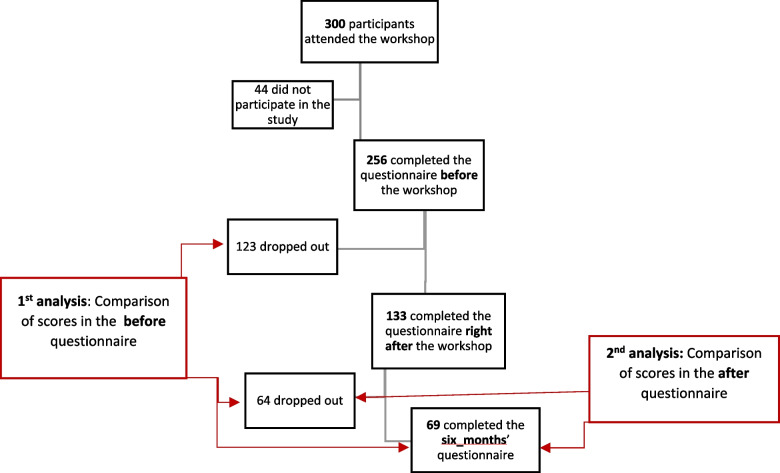
Schematic representation of self-selection bias analysis. The red arrows depict the groups tested in each analysis

Given the significant rate of non-completion of the follow up questionnaires, we tested the possibility of self-selection bias (Fig. 4). Two separate analyses were conducted using non-paired t-tests. In the first analysis, we compared the scores on the questionnaires collected before the workshop of the 69 participants who completed three questionnaires (before, right after and six months after) to the scores of the 123 students who completed only one (before) and of the 64 students who completed only two (before and right after). Statistical analysis did not reveal significant differences between the groups tested (*p* > 0.05). In the second analysis, we compared the scores on the questionnaires collected right after the workshop of the 69 participants who completed three questionnaires to those of 64 who completed only the before and right after questionnaires. There were no statistical differences between the groups (p > 0.05) in both cognitive and self-confidence questions, with the exception of question 6 where the group of 64 participants scored higher (*p* < 0.05).

### Analysis of all data

In this analysis we included all participants, 256 who completed the before questionnaire, 133 who completed the right after questionnaire and 69 who completed the six months after. The results were similar to the paired-sample analysis. Specifically, regarding the assessment of knowledge, the mean scores for the before, right after and six months after group were accordingly: 10.13 (SD ± 2), 11.77 (SD ± 1.53), 11.43 (SD ± 1.83). Three Wilcoxon signed-rank tests were performed comparing 1) right after vs before, 2) six months after vs before, and 3) six months after vs right after. The first two analyses revealed a statistically significant increase in knowledge right after and six months after the workshop compared to before (*p* < 0.0001). The third analysis revealed a statistically significant decrease in the score six months after compared to right after (*p* = 0.046). Regarding the assessment of self-confidence, statistically significant increases in scores were observed for all nine questions both in the right after and in the six months after group compared to before (*p* < 0.0001).

### Thematic analysis of comments

**Fig. 5:**
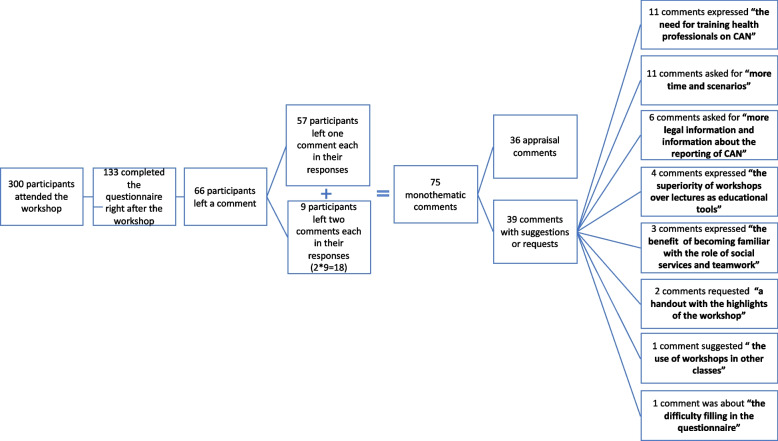
Flow-chart depicting the thematic analysis of participants’ comments

After the intervention, participants were asked to provide feedback or ideas regarding the workshop. Sixty-six participants of the 133 who completed questionnaires both before and after the workshop, chose to add a comment. Of these 66 participants, 57 raised only one issue per comment (monothematic comments) and the other nine raised two issues each in their responses. We chose to separately, twice-register, these nine responses depending on their topics, thus converting them to monothematic. After this conversion, 75 monothematic comments emerged. Of these 75 comments, 36 were simply positive about the workshop without any suggestions, while the remaining 39 also included suggestions. Eleven of these 39 cited the "need for training health professionals on the subject of CAN". Eleven participants requested "more time and more scenarios” in the workshop, while six other students requested "more legal information and more information about the reporting process of CAN". Four participants made a "comparison of workshops and lectures as educational tools" and they all claimed that workshops are more efficient due to their participatory nature. Three participants pointed out the benefit of becoming familiar with “the role of social services and teamwork”. Two participants requested a "handout with the highlights" of the workshop. One participant suggested the “use of workshops in other classes” of the medical school curriculum. Finally, one participant, after mentioning the commendable effort of the team, commented on his/her “difficulty filling in the questionnaire”, as he/she claimed that some questions of the questionnaire could not be answered based on the workshop (Fig. 5).

## Discussion

This study is the first to develop and evaluate an active learning approach for teaching CAN to medical students exclusively. Specifically, a series of participatory educational workshops with the use of peer-to-peer teaching, role playing with designated actors and mannikins with realistic depictions of physical findings was incorporated into the NKUA Medical School curriculum. To this effect, we successfully gathered, trained and established a peer-to-peer teaching group and participants showed significant gains in knowledge and increase in self-confidence in the recognition and management of CAN. Further studies are definitely needed to assess any long-term benefits of our educational intervention.

Contrary to most studies available in literature [15–17, 19–21], this workshop was designed and conducted exclusively for medical students. Since CAN is widespread in the general population, all physicians are expected to encounter at least one case of CAN during their career. Therefore, instead of focusing on training physicians of specific specialties, we opted to engage medical students to ensure that all medical graduates will possess basic skills and knowledge to recognize, evaluate and appropriately report suspected CAN. Adopting an interdisciplinary approach is crucial in the management of CAN [34], hence particular efforts were made to discuss the role of other health care professionals in the evaluation and management of CAN, including nursing staff and hospital and community social workers. Taking a detailed medical history while making sure that the described mechanism of injury is consistent with the child’s developmental stage and clinical status, performing a thorough clinical examination, requesting targeted laboratory and imaging studies and assessing them properly, identifying signs, symptoms and social indicators of CAN, recognizing the need for hospital admission, as well as making a referral to the investigative authorities when one’s level of suspicion is high are all necessary skills that healthcare students and professionals often report they lack [9–13, 17–20, 23, 35] and that we, therefore, attempted to teach our workshop’s participants.

The initial gains observed right after the workshop decreased in the six-month follow-up evaluation. However, even six months after the workshop, participants performed better than before, both in the cognitive questions and the self-assessment confidence questions. In addition, self-selection bias testing indicated that the entire sample was statistically homogenous, since the group of 69 participants who completed all three questionnaires was representative of the starting and each subsequent group. This conclusion is based on the increases in knowledge and self-confidence right after the workshop and six months after the workshop, that were observed in every comparison between each group of our starting sample.

Based on the qualitative data gathered, medical students expressed their enthusiasm and provided useful tips to help improve the conduction of the workshops. Medical student evaluations at the NKUA are not requested consistently, but, when provided, have the potential of influencing medical curriculum decisions. Although the medical curriculum is already overly filled and there was initial reluctance to allow two teaching hours on CAN, this participatory workshop has been highly rated by students and, therefore, has become a staple part of the clerkship in Pediatrics. The development of the workshops was based on expert recommendations on CAN education [36–40]. It has been suggested that certain social characteristics of the patients themselves and/or their families might affect identification, investigation and reporting of the possibility of CAN by physicians [41–43]. Therefore, an attempt to address certain stereotypes through carefully selected socially diverse case scenarios was made. Although this social component was briefly discussed in the workshops, it should be apparent that the present intervention's main goal was to instruct medical students on how to identify and manage CAN using a universal evidence-based approach. The implementation of specific strategies for systemic issues in medical education is still required.

Case scenarios included real life examples of sexual and physical abuse, neglect, and accident. The goal of the scenarios was to illustrate diagnostic uncertainties and the importance of determining the threshold to report child safety concerns. However, it was stressed that accidents do happen, and although preventive efforts should be made, overdiagnosis of CAN should be avoided.

A strength of our study was the use of questionnaires both short- and long-term to assess the effectiveness of the workshop on two important dimensions for practicing future physicians, knowledge, and confidence. Most students had not previously had any education on CAN, strengthening the results of our study. The improvement of self-confidence demonstrated in our study may enhance responsible practices related to CAN. We postulate that several features of this workshop may have contributed to the significant and long-lasting gain of knowledge and improvement of self-confidence for participants. First, several screening clinical tools were provided in a checklist form that participants were repeatedly encouraged to use. Checklists, though sometimes time-consuming and incomplete, are useful for codifying interventions, removing ambiguity, alerting clinical staff to the possibility of CAN and improving recording of important clinical information [19, 44, 45]. Second, as scenarios, mannikins and corresponding laboratory and imaging studies were derived from everyday clinical practice, the experience was rendered practical for participants. Third, engaging participants in the decision “to report or not to report” and discussing common pitfalls and facilitators regarding the reporting procedure, may have increased self-reported confidence in reporting suspected CAN.

Completion of the questionnaires themselves both right after and six months after the workshop may have boosted self-confidence and served as a reminder of the CAN issues discussed. In fact, it has been suggested that retrieval practice can facilitate long-term memory retention; much like repeated studying does, even without feedback [46].

There are at least six limitations of this study. The main limitation is the lack of a control group, rendering comparison of gain and retention of knowledge and self-confidence between the workshop and a traditional power-point lecture impossible. Indeed, a gold-standard evaluation method analogous to the ones used in clinical research, i.e., a Randomized Controlled Trial, would produce more firm outcomes. However, education research lacks gold-standard evaluation methods, since it is prone to errors that randomization cannot control for, some of those being variations in those implementing the intervention, high participant drop-out rates and other execution and contextual factors [47]. There are previous studies indicating that interactive, case-based learning is indeed more effective than a traditional didactic lecture [15–17, 48]. It should be noted that the use of a pre-test may have improved the post-test outcomes, making the workshop’s efficacy less apparent, but this effect itself seems to be dependent on participants’ characteristics and the quality of instruction [49]. Another limitation is the use of mannikins, thus precluding the practice by medical students of communicating with and examining children. Moreover, since all patients described in the scenarios were infants and toddlers, participants may have developed the false impression that only young children are affected by CAN. Although all cases were presented to the participants at the end of the workshop, time did not suffice for participants to manage more than one case scenario. Finally, attempts to consistently engage other professionals (nursing staff, hospital social workers) failed, limiting the strength of this educational program.

## Conclusion

In summary, we have demonstrated a feasible and novel active learning session on child abuse and neglect and have shown that this education improves both knowledge and self-confidence in a group of medical students. We are optimistic that CAN education will expand and be refined to include an increasing number of medical students, practicing clinicians and health care workers. However, the true impact of the workshop on detecting and reporting suspected CAN and ultimately safeguarding the lives and well-being of children in Greece lies in the future.

## Supplementary Information

**Additional file 1: Table 2.** Knowledge questions (modified from Soldatou et al., 2020 [18]).

**Additional file 2: Table 3.** Self-assessment questions (modified from Lee et al., 2012 [33]).

## Data Availability

Data available on request from the authors.

## Abbreviations

CAN: Child abuse and neglect
CANRSE: CAN reporting self-efficacy
GDPR: General Data Protection Regulation
NKUA: National and Kapodistrian University of Athens
SBME: Simulation-Based Medical Education
SD: Standard Deviation
TEN: Torso, Ears, Neck
WHO: World Health Organization
