# Supplementary material for: Creation and evaluation of a participatory child abuse and neglect workshop for medical students
Source: BMC Med Educ. 2022 Nov 16;22:797. doi: 10.1186/s12909-022-03837-2 (PMC9670524; doi:10.1186/s12909-022-03837-2)
Supplement: Supplementary file 1 — Additional file 1: Table 2. Knowledge questions (modified from Soldatou et al., 2020 [18]). [file 12909_2022_3837_MOESM1_ESM.docx]

| **Question 1** | The primary purpose for identifying and reporting child abuse should be to:   1. Protect the child and punish the parents. 2. Protect the child and preserve family integrity--- if possible. 3. Make an example for society. 4. Publicize the atrocity in the newspaper. 5. Obey the law. |
| --- | --- |
| **Question 2** | What is the most important question from the medical history, in order to identify child abuse?   1. Previous hospitalisations/ER visits 2. When was the child last seen normal? 3. Are there any (other) illnesses? 4. What is the family’s socio-economic status? |
| **Question 3** | Most children with failure to thrive have an organic basis for the difficulty.   1. True 2. False |
| **Question 4** | Rarely will parents who abuse their child attribute the actions to the child, a sibling, or baby-sitter.   1. True 2. False |
| **Question 5** | Parents do have a right to refuse treatment of their children.  The professional has a duty to assure proper medical care of children--- despite parents’ opinions.   1. True 2. False |
| **Question 6** | Neglect is best defined as:   1. Failure of parents to provide essential care to a child 2. Failure of parents to provide essential care to a child, resulting in serious harm 3. A child’s basic need is not met, regardless of the cause(s) 4. A child’s basic need is not met, resulting in serious harm 5. A child’s basic need is not met due to an intentional act by the parents |
| **Question 7** | Which of the following would be least useful in the child abuse medical evaluation of a 4-month-old with a spiral fracture of the humerus   1. Head CT without contrast 2. Ophthalmologic consultation 3. Thorough history from the caregiver 4. Babygram of child’s bones 5. Abdominal trauma lab panel (ALT, AST, Amylase, Lipase) |
| **Question 8** | Multiple preventable accidents such as burns, ingestions or falls may be reported as safety neglect.   1. True 2. False |
| **Question 9** | It is highly unlikely that a one-year-old child will suffer a fracture from falling out of bed or off a couch.   1. True 2. False |
| **Question 10** | Injuries that suggest child abuse:   1. Spiral fracture in a one-year-old, bilateral black eyes, cigarette burns, rib fractures. 2. Green stick fractures in a 4-year-old. 3. Crusted circular lesions, with one pustule, on the face of a two-year-old. 4. Blue patches on buttocks of one-week-old dark-skinned infant. |
| **Question 11** | Retinal hemorrhages and subdural hemorrhage in an infant suggest shaken-impact syndrome.   1. True 2. False |
| **Question 12** | The presence of multiple injuries of different ages is strong evidence for abuse.   1. True 2. False |
| **Question 13** | Inadequate or inappropriate explanation for a single injury should not suggest child abuse.   1. True 2. False |
| **Question 14** | Bilateral black eyes in a 2-year-old. Fell off tricycle. No other injuries   1. Story compatible with injury 2. Skull and long bone series needed 3. Suspect child abuse 4. Bleeding workup needed 5. b, c, d |
| **Question 15** | Geometric burns on both feet of a 6-month-old who “walked on a floor grid”.   1. Poor family 2. History compatible with injury 3. Neglect 4. Child abuse: injury incompatible with developmental level 5. None of the above |

Table 2. Knowledge questions (modified from Soldatou et al., 2020 [18])
