# Supplementary material for: Creation and evaluation of a participatory child abuse and neglect workshop for medical students
Source: BMC Med Educ. 2022 Nov 16;22:797. doi: 10.1186/s12909-022-03837-2 (PMC9670524; doi:10.1186/s12909-022-03837-2)
Supplement: Supplementary file 2 — Additional file 2: Table 3. Self-assessment questions (modified from Lee et al., 2012 [33]). [file 12909_2022_3837_MOESM2_ESM.docx]

| **Question 1** | “*I am confident that I can recognize suspected physical abuse cases*”. |
| --- | --- |
| **Question 2** | ” *I am confident that I can recognize suspected neglect cases*”. |
| **Question 3** | “*I am confident that I can recognize suspected sexual abuse cases*”. |
| **Question 4** | “*I am confident that I can report suspected physical abuse, neglect or sexual abuse cases*” |
| **Question 5** | “*I am confident that can ask for support from medical staff to report suspected physical abuse, neglect, or sexual abuse*” |
| **Question 6** | “*I am confident that I can interview a parent regarding suspected physical abuse, neglect or sexual abuse*” |
| **Question 7** | “*How confident are you that reporting suspected child physical abuse, neglect or sexual abuse is in the child’s best interest*”. |
| **Question 8** | “*How confident are you that reporting suspected child physical abuse, neglect or sexual abuse is in the family's best interest*”. |
| **Question 9** | “*How confident are you that the qualified Services will respond appropriately when reports of physical abuse, neglect or sexual abuse are made*”. |

**Table 3.** Self-assessment questions (modified from Lee et al., 2012 [33]).
